# Medication adherence in patients with type 2 diabetes after disability onset: a difference-in-differences analysis using nationwide data

**DOI:** 10.1186/s12916-024-03324-z

**Published:** 2024-03-06

**Authors:** Szu-Han Chen, Miaw-Chwen Lee, Christy Pu

**Affiliations:** 1 Department of Medicine, College of Medicine, National Yang Ming Chiao Tung University, Taipei, Taiwan; 2 Department of Social Welfare, National Chung Cheng University, Chia-Yi, Taiwan; 3 Center for Innovative Research on Aging Society, National Chung Cheng University, Chiayi, Taiwan; 4 Advanced Institute of Manufacturing with High-tech Innovations, National Chung Cheng University, Chiayi, Taiwan; 5 Institute of Public Health, College of Medicine, National Yang Ming Chiao Tung University, 155 Li-Nong ST, Sec 2, Peitou, Taipei, Taiwan

**Keywords:** Medication possession ratio, Medication adherence, Disability, Type 2 diabetes

## Abstract

**Background:**

Effectively managing the coexistence of both diabetes and disability necessitates substantial effort. Whether disability onset affects adherence to type 2 diabetes medication remains unclear. This study investigated whether disability onset reduces such adherence and whether any reduction varies by disability type.

**Methods:**

This study used the National Disability Registry and National Health Insurance Research Database from Taiwan to identify patients with type 2 diabetes who subsequently developed a disability from 2013 to 2020; these patients were matched with patients with type 2 diabetes without disability onset during the study period. Type 2 diabetes medication adherence was measured using the medication possession ratio (MPR). A difference-in-differences analysis was performed to determine the effect of disability onset on the MPR.

**Results:**

The difference-in-differences analysis revealed that disability onset caused a reduction of 5.76% in the 1-year MPR (*P* < 0.001) and 13.21% in the 2-year MPR (*P* < 0.001). Among all disability types, organ disabilities, multiple disabilities, rare diseases, and a persistent vegetative state exhibited the largest reductions in 2-year MPR.

**Conclusions:**

Policies aimed at improving medication adherence in individuals with disabilities should consider not only the specific disability type but also the distinct challenges and barriers these patients encounter in maintaining medication adherence.

## Background

According to the International Diabetes Federation, 537 million adults (i.e., 10.5% of the adult population worldwide) were living with diabetes in 2021 [1]. Type 2 diabetes accounts for 98% of global diabetes diagnoses [2]. It can lead to adverse patient outcomes, necessitating optimal adherence to diabetes medication [3, 4]; however, adherence remains an ongoing challenge [5]. The majority of patients fail to achieve their recommended glycemic goals, and this failure may be at least partly due to unsatisfactory adherence to therapeutic interventions [5].

In 2020, approximately 16.2% of individuals with disabilities in the United States were diagnosed as having diabetes, equating to approximately 1 in 6 people; by contrast, only 7.5%—or 1 in 14 people—without disabilities were diagnosed as having diabetes [6]. Although many studies have examined the reasons behind suboptimal adherence to diabetes medication and devised strategies to address them, few have focused on individuals with disabilities. The onset of a disability can cause new health-related and financial burdens, placing patients in a vulnerable position; this can cause poor adherence to medication for non-disability-related chronic diseases, especially by people with disadvantageous conditions, such as low income [7–9].

Special care is commonly required for individuals with disabilities. Unfortunately, people with disabilities and severe health conditions often encounter challenges in accessing proper medical services, despite often facing higher medical costs than their healthy counterparts [10–12].

Poor glycemic control in individuals with type 2 diabetes can lead to disabilities such as visual impairment or require limb amputation [13, 14]. Nevertheless, disability onset after type 2 diabetes can reduce type 2 diabetes medication adherence because both conditions require substantial medical treatments; it can become burdensome for an individual to simultaneously manage their multiple health conditions [15]. Patients with both disability and diabetes may prioritize managing their disability, particularly when they have access to limited resources. In addition, people with disabilities may be unable to adhere to nonpharmacological treatment recommendations for diabetes, such as exercise, because of their mental or physical limitations [16], thus increasing their reliance on oral or intravenous insulin is essential for glycemic control. Unlike individuals without an intellectual disability, those with this problem may face cognitive challenges in managing diabetes and its complexities [17]; these challenges reduce their medication adherence. To improve diabetes outcomes, the effect of disability onset on the medication adherence of patients with type 2 diabetes must be examined. Therefore, we analyzed the effect of disability onset on type 2 diabetes medication adherence. We hypothesized that disability onset would negatively affect medication adherence for type 2 diabetes.

## Methods

### Data sources

We used 2010–2020 data from Taiwan’ National Health Insurance (NHI) Research Database. The NHI is mandatory for all citizens and covers approximately 24 million individuals. The NHI database is overseen by the Ministry of Health and Welfare. It provides detailed information on both inpatient and outpatient services used within the NHI system, including drug reimbursement, and includes data for all individuals, regardless of whether they made any medical claims in a specific year [18].

To identify individuals with disabilities, we used the National Disability Registry from 2015 to 2020. Individuals who meet the criteria for disability status can receive various benefits, such as financial support, employment assistance, and tax exemptions, making them highly likely to be in the registry. Thus, we could confidently identify individuals with disabilities.

The National Disability Registry can be linked to NHI data by using anonymous individual-specific identification numbers. The data of all individuals analyzed in the current study were anonymized before being released to the researchers, and individual informed consent was waived. This study was approved by the Institutional Review Board of National Yang Ming Chiao Tung University (approval number: YM107047E).

### Definition of disability

In Taiwan, individuals who wish to obtain disability status must fill out an application specifying the type of disability they are seeking approval for, such as visual or limb disability. The evaluation is conducted by a specialist in the field of the disability being assessed. For example, an ophthalmologist or otolaryngologist evaluates a claim of visual or hearing impairment, respectively. The specialist assesses the applicant’s level of function related to the particular disability and determines whether they meet the criteria for the disability, by following a set of assessment guidelines for that disability. For instance, for assessments of limb disabilities, the guidelines recommend the evaluation of functional joint movement and muscle strength in all limbs, muscle tension, voluntary movement, and the structure abnormalities of the trunk and extremities.

The categories of disabilities recognized in Taiwan are visual disability, hearing disability, vocal disability, limb disabilities, intellectual disabilities, multiple disabilities, disabilities related to organ malfunction, facial disfigurement, a persistent vegetative state, dementia, autism, chromosomal abnormality, mental disabilities, motion or balance impairment, rare disease, and intractable epilepsy.

### Definition of type 2 diabetes

Patients with type 2 diabetes were identified using the *International Classification of Diseases, Ninth Revision, Clinical Modification* (*ICD-9-CM*) diagnostic codes 250.00, 250.02, 250.10, 250.12, 250.20, 250.22, 250.30, 250.32, 250.40, 250.42, 250.50, 250.52, 250.60, 250.62, 250.70, 250.72, 250.80, 250.82, 250.90, and 250.92 and *International Classification of Diseases*, *Tenth Revision*, *Clinical Modification* (*ICD-10-CM*) diagnostic codes E11. Type 2 diabetes was defined as ≥ 1 outpatient claim with one of the aforementioned codes and a concurrent prescription for diabetes medication. Including patients with drug prescriptions was necessary to exclude patients with a type 2 diabetes code for checkup purposes or “ruling-out” the condition.

### Study patients

We included patients whose first disability record appeared in the National Disability Registry in or after 2015 because, in 2012, the Taiwanese government mandated that individuals with a disability status update their registry status, even if they had already been granted permanent disability status. This requirement resulted in a surge of registry renewals in 2013–2014. Consequently, we focused on only those individuals whose first disability records appeared in or after 2015 to ensure that each record in our data represented a newly approved disability rather than a renewal of a previous one.

We conducted a difference-in-differences (DID) analysis. The inclusion criteria were as follows: having type 2 diabetes for at least 2 years before and after disability onset and being alive at least till the end of the 4-year study period. Because the most recent year with National Disability Registry data was 2020, we excluded patients with disability onset after 2018 (to ensure the 2-year period after onset). We then identified patients who had received type 2 diabetes diagnoses ≥ 2 years before their disability onset and did not die within 2 years after disability onset. Finally, we enrolled 339,387 patients. These patients were then age- and sex-matched in a 1:2 ratio with patients with type 2 diabetes but without disabilities during the study period.

### Medication adherence

We used the medication possession ratio (MPR), a widely used tool to measure diabetes medication adherence [19]. MPR is calculated by dividing the total number of days for which a patient has medication by the number of days for which the patient is recommended to have the medication.

MPR was calculated using patients’ NHI claims data, particularly their outpatient records. These records provided information on exact outpatient visit dates in addition to corresponding diagnostic codes (*International Classification of Diseases*, *Tenth Revision*, *Clinical Modification*). By linking these records to patients’ medication history data, we identified the medicines prescribed during each outpatient visit. Moreover, information pertaining to pharmacy refills was collected from the data set. Medication data were analyzed to determine the duration of medication use. A patient may be prescribed multiple diabetes medications during a single visit. In such cases, the medication with the longest prescribed duration determines the number of days used for each visit.

We defined the denominator as 365 minus the number of hospitalization days because the number of days of drug prescriptions are not available in NHI inpatient records. This, however, should not have significantly altered our results, because the number of hospitalization days was low for most patients.

We used the Anatomical Therapeutic Chemical (ATC) classification to identify both oral and injectable hypoglycemic drugs for type 2 diabetes. The first 5 characters of the ATC codes used were A10AB, A10AC, A10AD, A10AE, A10BA, A10BB, A10BF, A10BG, A10BX, A10BH, A10BJ, and A10BK. We then identified the corresponding drugs by using the NHI Drug Item System. The complete list of drugs considered in this study is available upon request.

Because the MPR is dependent on the period, we calculated 3 sets of MPRs for analysis [8]: (1) MPRs 1 year before and 1 year after disability onset (1-year MPRs), (2) MPRs 2 years before and 2 years after disability onset (2-year MPRs, excluding the 1-year MPRs), and (3) MPRs averaged over the first and second years before and after disability onset (2-year average MPRs over a 730-day period).

### Statistical analysis

To create a control group, we used the DID method. The DID method is often used for causal inference in medical and public health studies [20]. The index date was defined as the earliest date of disability diagnosis, as indicated by data from the National Disability Registry. For control patients, that is, those without disabilities, the index date was the same as that of their matched counterpart with disabilities. For a prescription that spanned across the index date, we calculated the number of days the medication was prescribed both before and after the index date. Using the *t* test, we first compared MPRs calculated using different definitions before and after the index date. Then, MPR was modeled using the following equation [21].

$${MPR}_{i}={b}_{1}+{b}_{2}{disability}_{i}+{b}_{3}{Post}_{i}+{b}_{4}{(disability\times Post)}_{i}+x\beta +{\epsilon }_{i}$$

Linear regression was used. Here, *Post*_*i*_ is a dummy variable indicating the period after the index date for individual *i*, and $x\beta$ is a set of covariates. ${b}_{4}$ is the estimated effect of disability on the MPR. Inverse probability treatment weight for having a disability was calculated through logistic regression; the resulting value was incorporated into the regression model. A separate regression was performed for each disability type. In each regression, we controlled for sex, area of residence (e.g., the Taipei, North, Central, South, and Kao–Ping regions), insurable income under the NHI (tertiles), and low-income status (yes or no). All statistical analyses were conducted using STATA 15 (College Station, TX, USA) [22].

## Results

### Sociodemographic characteristics

Table 1 presents the baseline sociodemographic characteristics of the patients with (*n* = 120,075) and without (*n* = 240,150) disabilities. Because the patient groups were matched by age, sex, income tercile, and low-income status, no significant between-group difference was observed in the distribution of any of these variables.

**Table 1:** Patient characteristics

	Without disability (*n* = 240,150)	With disability (*n* = 120,075)	*P*-value
Age (mean ± SD)	73.32 ± 0.04	73.32 ± 0.03	1.000
Sex			1.000
Male	126,994 (52.88%)	63,497 (52.88%)	
Female	113,156 (47.12%)	56,578 (47.12%)	
Area			1.000
Taipei	36,012 (15.00%)	18,006 (15.00%)	
North	65,964 (27.47%)	32,982 (27.47%)	
Central	43,036 (17.92%)	21,518 (17.92%)	
South	80,306 (33.44%)	40,153 (33.44%)	
Kao-Ping, East, and others	14,832 (6.18%)	7416 (6.18%)	
Income quantile			1.000
Tercile 1	72,734 (30.29%)	36,367 (30.29%)	
Tercile 2	99,046 (41.24%)	49,523 (41.24%)	
Tercile 3	68,370 (28.47%)	34,185 (28.47%)	
Low-income status			1.000
Yes	238,444 (99.29%)	119,222 (99.29%)	
No	1706 (0.71%)	853 (0.71%)	

*SD* Standard deviation

### MPR before and after disability onset

**Fig. 1:**
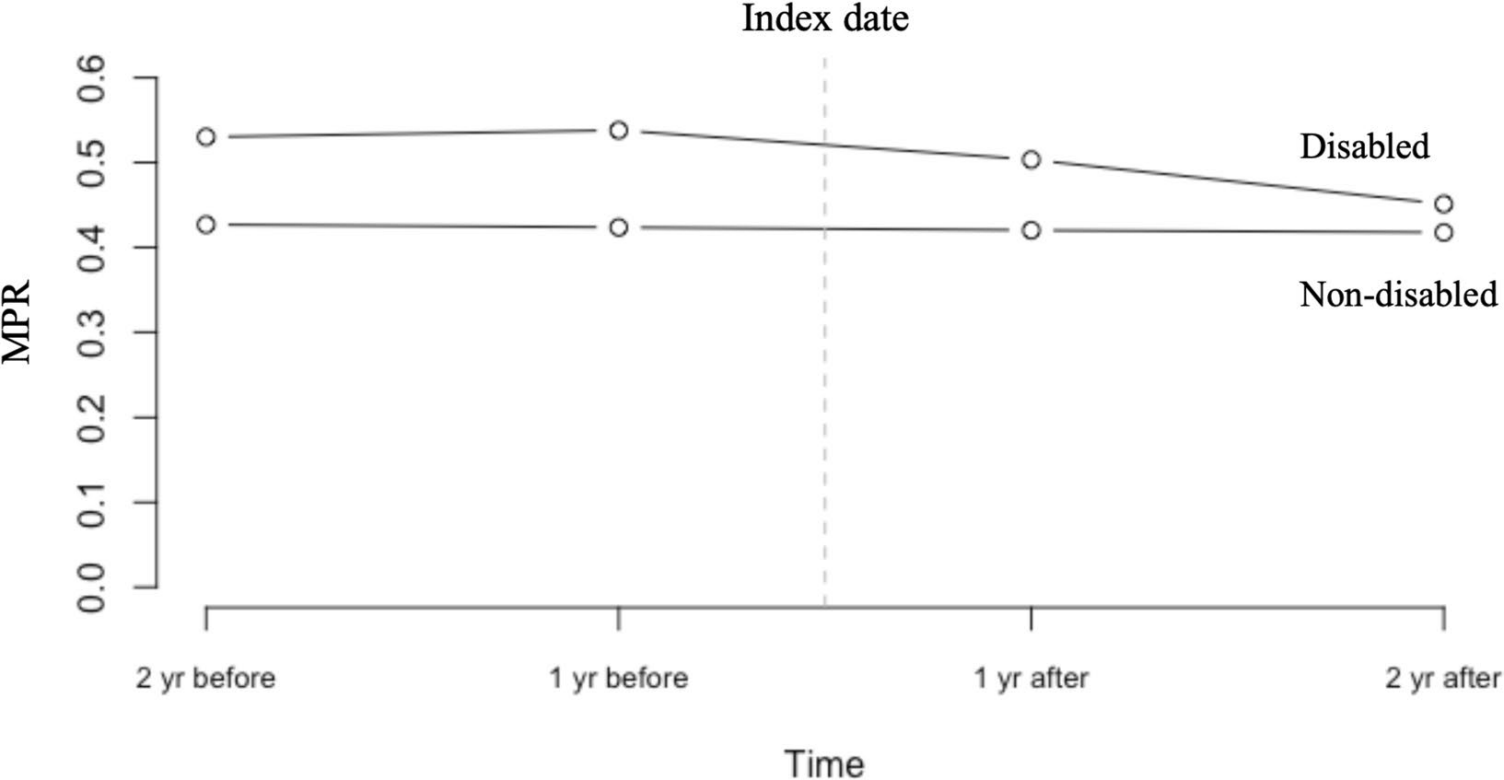
MPR before and after index date for people with and without disability

Figure 1 and Table 2 presents the MPRs of the patients with and without disabilities before and after the index date. The patients without disabilities had lower MPRs (all 3 types) before the index date than did those with disabilities (all *P* < 0.001). The average 2-year MPRs before the index date was 0.524 (95% confidence interval (CI): 0.522–0.526) and 0.414 (95% CI: 0.412–0.415) in the patients with and without disabilities, respectively. However, the MPRs (all 3 types) of the patients with disabilities decreased after disability onset.

**Table 2:** MPR before and after onset of disability

	Before index date			After index date		
	*n*	Mean (95% CI)	MPR definitions	*n*	Mean (95% CI)	*p*-value*
With disability			With disability			
MPR 2 year	120,075	0.530 (0.528–0.533)	MPR 1 year	120,075	0.503 (0.501–0.506)	< 0.001
MPR 1 year	120,075	0.538 (0.536–0.540)	MPR 2 year	120,075	0.451 (0.449–0.454)	< 0.001
MPR 2-year average	120,075	0.524 (0.522–0.526)	MPR 2-year average	120,075	0.466 (0.464–0.469)	< 0.001
Without disability			Without disability			
MPR 2 year	240,150	0.427 (0.425–0.429)	MPR 1 year	240,150	0.420 (0.418–0.422)	< 0.001
MPR 1 year	240,150	0.424 (0.422–0.425)	MPR 2 year	240,150	0.418 (0.416–0.419)	< 0.001
MPR 2-year average	240,150	0.414 (0.412–0.415)	MPR 2-year average	240,150	0.407 (0.405–0.408)	< 0.001

*CI* Confidence interval

^*^Before vs. after the index date

Table 3 presents the MPRs before and after the index date for the patients with disabilities, stratified by disability type. The MPRs varied by disability type (*P* < 0.001). During the study period, patients with visual, hearing, or organ disability had the highest MPRs before the index date, whereas those with epilepsy or mental disability had relatively low MPRs.

**Table 3:** Disability types and changes in MPR

	Before index date				After index date				
		2-year MPR	1-year MPR	2-year average MPR			1-year MPR	2-year MPR	2-year average MPR
	*n*	Mean (95% CI)	Mean (95% CI)	Mean (95% CI)		*n*	Mean (95% CI)	Mean (95% CI)	Mean (95% CI)
Visual	5854	0.584 (0.573, 0.595)	0.635 (0.624, 0.645)	0.595 (0.585, 0.605)		5854	0.643 (0.633, 0.654)	0.611 (0.600, 0.622)	0.611 (0.602, 0.621)
Hearing	11,125	0.534 (0.525, 0.542)	0.544 (0.536, 0.552)	0.525 (0.517, 0.533)		11,125	0.553 (0.544, 0.561)	0.542 (0.534, 0.550)	0.533 (0.526, 0.541)
Verbal	1235	0.432 (0.408, 0.457)	0.479 (0.455, 0.503)	0.445 (0.423, 0.468)		1235	0.448 (0.423, 0.472)	0.408 (0.383, 0.433)	0.417 (0.394, 0.439)
Limb	30,423	0.475 (0.470, 0.480)	0.505 (0.500, 0.510)	0.481 (0.476, 0.485)		30,423	0.494 (0.489, 0.499)	0.434 (0.429, 0.439)	0.453 (0.449, 0.458)
Intellectual	366	0.276 (0.236, 0.317)	0.322 (0.280, 0.364)	0.291 (0.253, 0.330)		366	0.398 (0.355, 0.442)	0.453 (0.408, 0.498)	0.412 (0.372, 0.453)
Multiple	11,437	0.501 (0.493, 0.509)	0.517 (0.509, 0.525)	0.501 (0.493, 0.508)		11,437	0.461 (0.453, 0.469)	0.370 (0.362, 0.378)	0.407 (0.400, 0.415)
Organ	35,897	0.608 (0.603, 0.612)	0.583 (0.579, 0.588)	0.585 (0.581, 0.589)		35,897	0.500 (0.495, 0.504)	0.449 (0.444, 0.453)	0.464 (0.460, 0.468)
Facial	132	0.427 (0.353, 0.501)	0.476 (0.402, 0.551)	0.440 (0.370, 0.509)		132	0.476 (0.391, 0.450)	0.447 (0.369, 0.525)	0.451 (0.377, 0.525)
Vegetative state	799	0.464 (0.433, 0.495)	0.495 (0.464, 0.525)	0.475 (0.446, 0.504)		799	0.420 (0.124, 0.148)	0.269 (0.242, 0.297)	0.340 (0.314, 0.366)
Dementia	17,107	0.538 (0.531, 0.544)	0.540 (0.533, 0.546)	0.527 (0.521, 0.533)		17,107	0.514 (0.507, 0.520)	0.447 (0.440, 0.454)	0.469 (0.463, 0.475)
Autism	10	0.193 (-0.084, 0.469)	0.197 (-0.100, 0.495)	0.190 (-0.090, 0.471)		10	0.180 (− 0.092, 0.452)	0.134 (− 0.426, 0.734)	0.151 (− 0.065, 0.367)
Chromosomal	36	0.493 (0.338, 0.648)	0.480 (0.337, 0.623)	0.475 (0.334, 0.616)		36	0.600 (0.457, 0.743)	0.580 (0.426, 0.734)	0.572 (0.438, 0.706)
Mental	4029	0.317 (0.304, 0.330)	0.353 (0.340, 0.366)	0.330 (0.318, 0.407)		4029	0.394 (0.380, 0.407)	0.413 (0.400, 0.427)	0.400 (0.388, 0.412)
Balance	679	0.498 (0.465, 0.532)	0.516 (0.484, 0.548)	0.498 (0.468, 0.529)		679	0.522 (0.490, 0.555)	0.478 (0.444, 0.512)	0.489 (0.459, 0.519)
Epilepsy	83	0.296 (0.206, 0.387)	0.356 (0.212, 0.450)	0.318 (0.232, 0.403)		83	0.428 (0.332, 0.523)	0.419 (0.327, 0.511)	0.410 (0.324, 0.496)
Rare disease	3	0.327 (− 1.080, 1.734)	0.255 (− 0.843, 1.353)	0.278 (− 0.335, 0.892)		3	0.005 (− 0.018, 0.029)	0.170 (− 0.561, 0.901)	0.084 (− 0.261, 0.430)
Others	860	0.461 (0.431, 0.491)	0.470 (0.440, 0.499)	0.457 (0.429, 0.485)		860	0.461 (0.431, 0.490)	0.424 (0.395, 0.453)	0.434 (0.408, 0.460)

### DID estimation

The DID estimate is presented in Table 4. Without stratification by disability type, disability onset caused 5.76% (0.031/0.538*100) reduction in 1-year MPR (*P* < 0.001) and 13.21% (0.070/0.530*100) reduction in 2-year MPR (*P* < 0.001). Substantial reductions were noted in the MPRs of those with multiple or organ disability (approximately 24% reduction in 2-year MPRs for both types of disabilities). Visual and limb disabilities may or may not be caused by type 2 diabetes. For limb disabilities, 2-year MPR decreased by 6.53%, after disability onset.

**Table 4:** Changes in MPR before and after onset of disability, DiD estimates

		MPR 2-year-average			MPR 1 year			MPR 2 year		
Disability type	*n*	Coefficient	95% confidence interval	*P*-value	Coefficient	95% confidence interval	*P*-value	Coefficient	95% confidence interval	*P*-value
Visual	5854	0.023	(0.008, 0.038)	0.003	0.012	(− 0.004, 0.029)	0.145	0.036	(0.020, 0.052)	< 0.001
Hearing	11,125	0.012	(< 0.001, 0.024)	0.050	0.015	(0.004, 0.026)	0.008	0.018	(0.006, 0.030)	0.004
Verbal	1235	− 0.022	(− 0.055, 0.011)	0.196	− 0.027	(− 0.063, 0.008)	0.129	− 0.015	(− 0.050, 0.021)	0.417
Limb	30,423	− 0.021	(− 0.028, − 0.014)	< 0.001	− 0.007	(− 0.015, < 0.001)	0.053	− 0.031	(− 0.039, − 0.024)	< 0.001
Intellectual	366	0.128	(0.068, 0.189)	< 0.001	0.079	(0.015, 0.144)	0.016	0.186	(0.121, 0.250)	< 0.001
Multiple	11,437	− 0.086	(− 0.097, − 0.075)	< 0.001	− 0.052	(− 0.064, − 0.041)	< 0.001	− 0.121	(− 0.133, − 0.109)	< 0.001
Organ	35,897	− 0.114	(− 0.121, − 0.107)	< 0.001	− 0.080	(− 0.087, − 0.073)	< 0.001	− 0.149	(− 0.156, − 0.142)	< 0.001
Facial	132	0.018	(− 0.082, 0.119)	0.721	0.003	(− 0.105, 0.111)	0.959	0.030	(− 0.078, 0.138)	0.588
Vegetative state	799	− 0.128	(− 0.169, − 0.087)	< 0.001	− 0.071	(− 0.145, − 0.027)	0.002	− 0.185	(− 0.229, − 0.141)	< 0.001
Dementia	17,107	− 0.051	(− 0.060, − 0.042)	< 0.001	− 0.022	(− 0.032, − 0.013)	< 0.001	− 0.081	( − 0.091, − 0.071)	< 0.001
Autism	10	− 0.032	(− 0.397, 0.333)	0.864	− 0.014	(− 0.406, 0.378)	0.945	− 0.049	(− 0.441, 0.342)	0.805
Chromosomal	36	0.104	(− 0.089, 0.296)	0.290	0.123	(− 0.083, 0.330)	0.242	0.097	(− 0.110, 0.303)	0.359
Mental	4029	0.077	(0.059, 0.096)	< 0.001	0.045	(0.025, 0.064)	< 0.001	0.106	(0.086, 0.126)	< 0.001
Balance	679	− 0.002	(− 0.047, 0.042)	0.921	0.010	(− 0.038, 0.057)	0.696	− 0.011	(− 0.058, 0.037)	0.657
Epilepsy	83	0.100	(− 0.027, 0.226)	0.124	0.076	(− 0.060, 0.212)	0.276	0.132	(− 0.004, 0.268)	0.057
Rare disease	3	− 0.187	(− 0.854, 0.480)	0.583	− 0.246	(− 0.962, 0.469)	0.500	− 0.148	(− 0.863, 0.568)	0.686
Others	860	− 0.016	(− 0.055, 0.024)	0.431	− 0.005	(− 0.048, 0.037)	0.800	− 0.028	(− 0.070, 0.015)	0.200
All disability	120,075	− 0.051	(− 0.055, − 0.047)	< 0.001	− 0.031	(− 0.036, − 0.027)	< 0.001	− 0.070	(− 0.074, − 0.065)	< 0.001

## Discussion

In this study, we analyzed the effect of disability onset on medication adherence in patients with type 2 diabetes. The following key findings were obtained: (1) medication adherence for type 2 diabetes significantly decreased after disability onset and (2) the magnitude of the decrease differed by disability type.

Understanding the negative effects of disability onset on type 2 diabetes medication adherence is crucial in the design of effective management policies and support systems [23]. Our study had several strengths. First, because we used population-based data, selection bias was not a threat. Second, because we used robust disability measurements, our findings are likely to be accurate and representative of the population with disabilities in Taiwan. Third, our data enabled us to analyze a wide range of disability types; such analysis is rare in the literature.

As life expectancy continues to rise globally, more older adults are living with both type 2 diabetes and disabilities, which can negatively affect their health-related quality of life [24, 25]. Adequate management of both conditions is imperative for ensuring the most favorable overall outcomes.

This study discovered that under the NHI, which ostensibly eliminated most financial barriers to medical access, people with newly onset disabilities still experienced a decrease in medication their relative adherence to that of their counterparts without disabilities. There are some plausible explanations. First, depending on the type and severity of the disability, individuals may face physical challenges and nonfinancial impediments to their access to pharmacies or health-care facilities. Costs related to special transportation can create additional financial burden for people with disabilities [26]. Second, some people with disabilities may rely on family members or other caregivers for support in managing their health, but such caregivers may not have adequate awareness of the patient’s health condition [27].

We observed that the reduction in MPR differed by disability type, with patients with organ disabilities or multiple disabilities exhibiting relatively high MPR reductions. These 2 disability types are more likely to be fatal than are other disabilities, causing patients or their caregivers to prioritize the allocation of their limited resources to managing these disabilities rather than to managing type 2 diabetes, which may be less clinically dominant or symptomatic. Furthermore, physicians of different specialties may provide conflicting advice to a patient if they consider one condition to be more severe. This underscores the importance of coordinated care for patients with type 2 diabetes facing other health conditions [28, 29].

Certain modifiable factors, such as a patient’s treatment beliefs, physician–patient communication, and dosage form (e.g., hypoglycemic agents vs. insulin), can be used to improve medication adherence [30, 31]. Our findings highlight the need to modify these factors for individuals with disabilities. For instance, patients with disabilities are more likely to perceive patient—physician communication as inadequate than are those without disabilities; therefore, physician–patient communication should incorporate disability literacy [32]. Future studies should analyze the mechanism underlying the adherence decline discovered in this study.

This study has several limitations. First, because of renewal mandates for disability registration, we could analyze the data of only those individuals with new disability onset in or after 2015, thus limiting the follow-up duration. Second, we used the National Disability Registry to identify disabilities; therefore, the definitions of disabilities may differ from those used in other circumstances. For example, according to our study, a person with an amputated limb would qualify for limb disability status; however, such an individual may not necessarily have limitations in their activities of daily living. Results based on different disability definitions may not be directly comparable with ours. Third, the NHI claims data only enabled the examination of adherence based only on MPR. Future studies should consider other adherence measures.

## Conclusion

Disability onset can adversely influence the medication adherence of patients with type 2 diabetes. Policies intended to enhance medication adherence among individuals with both diabetes and disabilities should consider the specific disability type and unique challenges faced by such patients in maintaining high medication adherence.

## Data Availability

The data that support the findings of this study are available from the Taiwan Ministry of Health and Welfare, but restrictions apply to available from the Taiwan Ministry of Health and Welfare, but restrictions the availability of these data, which were used under license for the current study, and so are not publicly available. Data are however available from the authors upon reasonable request and with permission of the Taiwan Ministry of Health and Welfare.

## Abbreviations

MPR: Medication possession ratio
NHI: National Health Insurance
ICD-9-CM: International Classification of Diseases, Ninth Revision, Clinical Modification
ICD-10-CM: International Classification of Diseases, Tenth Revision, Clinical Modification
DID: Difference-in-differences
ATC: Anatomical Therapeutic Chemical
